# *Pistacia lentiscus* extract enhances mammary epithelial cells’ productivity by modulating their oxidative status

**DOI:** 10.1038/s41598-020-78065-z

**Published:** 2020-12-02

**Authors:** O. Hadaya, R. Bransi-Nicola, Y. Shalev, H. Azaizeh, Z. Roth, H. Muklada, T. Deutch, S. Y. Landau, N. Argov-Argaman

**Affiliations:** 1 Department of Animal Sciences, Robert H. Smith Faculty of Agriculture, Food and Environment, The Hebrew University of Jerusalem, 7610001 Rehovot, Israel; 2 Department of Natural Resources, Institute of Plant Sciences, Agricultural Research Organization – the Volcani Center, 7505101 Rishon LeZion, Israel; 3 The Institute of Applied Research (Affiliated With University of Haifa), The Galilee Society, 20200 Shefa-Amr, Israel

**Keywords:** Lipids, Membrane trafficking

## Abstract

We assessed the potential of phenolic compounds from *Pistacia lentiscus* (lentisk) to enhance production of milk constituents in bovine mammary epithelial cells (MEC). MEC were exposed to 0 (control), 1 or 10 ppm of polyphenols from lentisk ethanolic extract (PLEE) for 24 h. PLEE were absorbed by the MEC plasma membrane, but also penetrated the cell to accumulate in and around the nucleus. PLEE increased triglyceride content in the cell and its secretion to the medium, and significantly increased intracellular lipid droplet diameter. Compared to control, PLEE increased dose-dependently the lactose synthesis, secretion of whey proteins, and contents of casein. To evaluate mitochondrial activity under pro-oxidant load, MEC were preincubated with PLEE and exposed for 2 h to H_2_O_2_. Exposure to H_2_O_2_ increased the proportion of cells with impaired mitochondrial membrane potential twofold in controls, but not in PLEE-pre-treated cells. Accordingly, proton leakage was markedly decreased by PLEE, and coupling efficiency between the respiratory chain and ATP production was significantly enhanced. Thus, lentisk polyphenols divert energy to production of milk fat, protein and lactose, with less energy directed to cellular damage control; alternatively, PLEE enables MEC to maintain energy and oxidative status under extreme metabolic rate required for milk production and secretion, and reduces the limitation on energy required to support production.

## Introduction

Selection of dairy animals for production traits results in constant physiological and metabolic stress in various organs and tissues, and especially in the mammary gland^1^. Metabolic stress is often manifested in the production and accumulation of reactive oxygen species (ROS), as demonstrated in rat lactating mammary glands^2^ and in mammary gland epithelial cells (MEC) of lactating dairy cows^3^.

ROS are the product of cellular physiological processes, primarily oxidative phosphorylation in the mitochondria, which are the main producer of cellular ROS^4^. These unstable molecules are important metabolites for normal physiological activity in mammalian cells, including signal-transduction cascades and homeostasis^5^. However, when ROS production exceeds the antioxidant capacity of the cell, ROS and other reactive metabolites accumulate, disrupting the redox status of the cell, and eventually resulting non-enzymatic oxidation of biological molecules, modification of nucleic acids and proteins, and lipid peroxidation and consequently, cellular dysfunction^6^. In addition, high levels of ROS may dysregulate cell division and tissue differentiation^7^, and disrupt the oxidative phosphorylation pathway^8^, resulting in reduced mitochondrial energy production. In the mammary gland, excess accumulation of ROS was associated with induced apoptosis in luminal, but not basal cells^9^. Also, excess accumulation of ROS can potentially impair the energy metabolism^4^. In the mammary gland, impaired energy metabolism is expected to reduce production and secretion of milk components, however this aspect and the underlying mechanism is yet to be determined.

Mammalian cells utilize two systems to mitigate ROS accumulation and restore redox status: (i) endogenous antioxidants such as glutathione, ubiquinol and bilirubin, and expression of antioxidant enzymes such as superoxide dismutase, catalase and glutathione peroxidase which are devoted to ROS detoxification^10^, and (ii) utilization of exogenous antioxidant compounds such as vitamin E and phenolic compounds to absorb the additional electrons or donate protons to reactive metabolites such as ROS, thereby quenching reactivity and stopping the propagation step of lipid peroxidation^11^.

The capacity to neutralize ROS by genetic (gene expression) or metabolic responses requires a ROS-sensing mechanism. This cellular mechanism relies on the repression and activation of the main cellular sensor of ROS, nuclear factor erythroid 2-related factor 2 (Nrf2). Oxidative signals release Nrf2 from its cytoplasm anchor, enabling its translocation into the nucleus to enhance the expression of genes with antioxidant response element in their promoter region^12^, including the enzymes sodium oxide dismutase and glutathione peroxidase. Moreover, to maintain “redox homeostasis”, those enzymes utilize reducing elements, such as NADPH, as electron and hydrogen donors^13^. Therefore, the activation of this mechanism can direct elements such as NADPH to maintain redox status, instead of performing their role in lactose synthesis and glycolysis. An alternative strategy for scavenging ROS is through the use of exogenous antioxidant resources, for example, dietary phenolic compounds which can be acquired from a dietary plant source^14^. Some of these plant secondary metabolites, if frequently consumed, maintain high concentrations of polyphenols in different organs^15^, which may alleviate the detrimental effects of an acute increase in ROS level. The mechanism by which phenolic compounds affect cellular ROS production is not entirely clear. Polyphenols might affect ROS by direct neutralization or by activation of the Nrf2 system, enhancing the cells’ capacity to quench ROS and other reactive metabolites^16,17^.

The use of plant extracts to mitigate inflammation has been known for thousands of years in human and veterinary medical practice^18^, and the Mediterranean evergreen *Pistacia lentiscus* (lentisk) is commonly used in traditional human and veterinary medicine^19^ due to its antimicrobial and antimutagenic activity^20,21^, antioxidant capacity^22,23^, and anticancer^24,25^, antifungal and antiparasitic^19^ properties. In dairy goats provided with *P. lentiscus* as a major source of forage, milk production and quality were much improved^26,27^. While some effects of *P. lentiscus*, such as elevated protein concentration in milk, can be explained by the polyphenol effect on rumen fermentation, digestion and absorption of proteins^28^, others, such as higher milk-fat production and phospholipid concentration in milk are most likely due to systemic effects of the *Pistacia* polyphenols^29^. Although lentisk has been widely investigated due to its mitigating effects on inflammation, to the best of our knowledge, the antioxidant role of *P. lentiscus* in mammary cell production traits has never been studied.

We designed an in-vitro model to assess the potential of *P. lentiscus* phenolic compounds for enhancing production of milk components in MEC and to investigate the involvement of mitochondrial activity in this process. Understanding this mechanism might lead to the development of new nutritional strategies for a further use of polyphenolic compounds to mitigate oxidative stress in ruminants.

## Results

Doses of PLEE used in the present study were according to preliminary dose response experiment with 1, 10 and 100 ppm of PLEE, which resulted in 99.01% ± 12.8 and 100.52% ± 29.4 and 40.9% ± 33.3 live cells, respectively, compared to 100% living cells in control group. Doses were chosen according to their toxic effect on live cell percentage after 24 h of treatment and according to solubility in the medium.

### *P. lentiscus* polyphenols penetrate MEC in culture and increase antioxidant capacity

**Figure 1:**
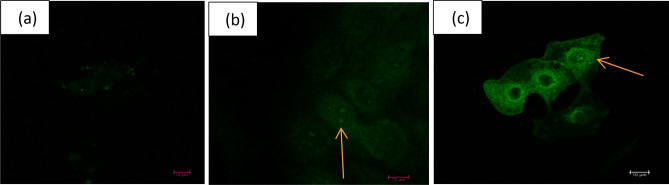
Confocal laser microscopy images of MEC without (**a**) or treated with 1 (**b**) or 10 (**c**) ppm PLEE for 24 h. Images show polyphenol penetration of MEC and the nucleus, as evidenced by their autofluorescence. The magnification power of the objective was × 63.

To investigate the association between polyphenols and MEC, the cells were exposed to 1 and 10 ppm PLEE. After exposure, cells were washed and visualized by confocal microscopy, taking advantage of the auto-fluorescence of polyphenols. The polyphenols penetrated the MEC cytoplasm and nucleus and gathered around the nuclei (Fig. 1). The autofluorescence intensity was higher for the 10 ppm vs. 1 ppm PLEE treatment, whereas no autofluorescence was observed in control cells, which were not exposed to the plant extract.

**Figure 2:**
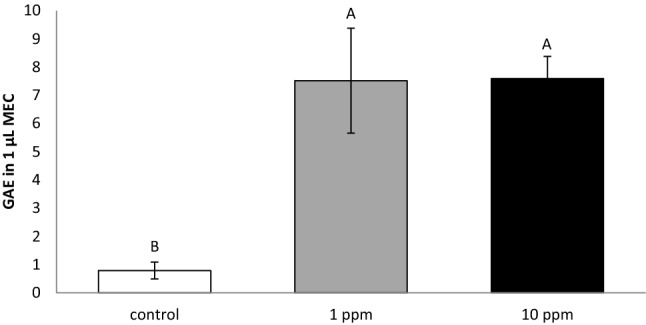
Antioxidant capacity of MEC incubated without (control, white) or with 1 (grey) or 10 (black) ppm PLEE for 24 h. Antioxidant capacity was analysed by chemiluminescence assay; n = 4 for each replicate in each treatment. Different letters indicate significant difference at *P* ≤ 0.05. GAE, gallic acid equivalent (µmol/L).

After exposure to the different PLEE concentrations, MEC were washed to remove any unbound extract, and the cells’ antioxidant capacity was determined by luminol-dependent chemiluminescence (LDCL) assay (Fig. 2). A ninefold increase in antioxidant capacity was found in treated cells (*P* = 0.046) compared to the untreated controls.

### P. lentiscus polyphenols increase MEC productivity

#### PLEE treatment alters lipid droplet size

**Figure 3:**
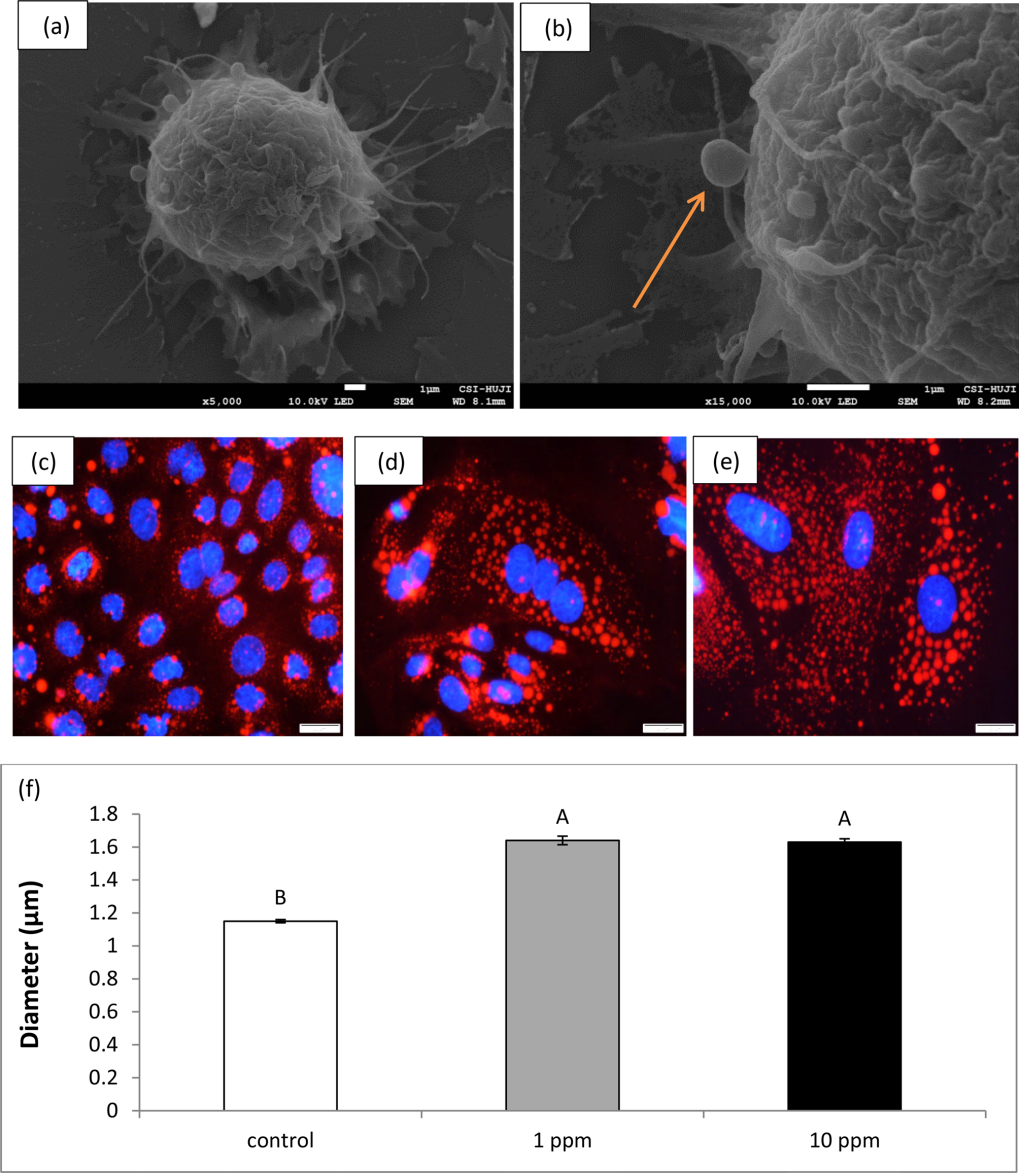
Primary bovine MEC milk fat globule secretion. Representative images on a glass surface at two magnifications, × 5000 (**a**) and × 15,000 (**b**), illustrate the activity of fat sprouting from the apical membrane of the MEC (indicated by arrow). Intracellular lipid droplet size was measured in MEC incubated without (control, white) or with 1 (grey) or 10 (black) ppm PLEE for 24 h. Representative images show control (**c**), 1 ppm (**d**) and 10 ppm (**e**) cells stained with DAPI (blue, nucleus) and Nile red (red, lipid droplets). Magnification resolution was × 20. Intracellular lipid droplet mean diameter was determined (**f**); n = 3 for each replicate in each treatment, with more than 2000 milk fat globules counted in each treatment. Different letters indicate significant difference at *P* ≤ 0.05.

Bovine MEC images were taken by scanning electron microscope (SEM), in order to document the secretion of fat globules (Fig. 3a,b). To determine whether PLEE treatment intervenes in the regulation of lipid droplet size, after 24 h of exposure to 1 or 10 ppm PLEE, MEC were stained with Nile red to measure the mean diameter of intracellular lipid droplets (Fig. 3c–e), images were taken by fluorescence microscopy. MEC treated with 1 or 10 ppm PLEE had 0.49 and 0.48 µm larger milk fat globules, respectively, than controls (Fig. 3f; P < 0.0001).

#### PLEE treatment alters lipid production and secretion

**Figure 4:**
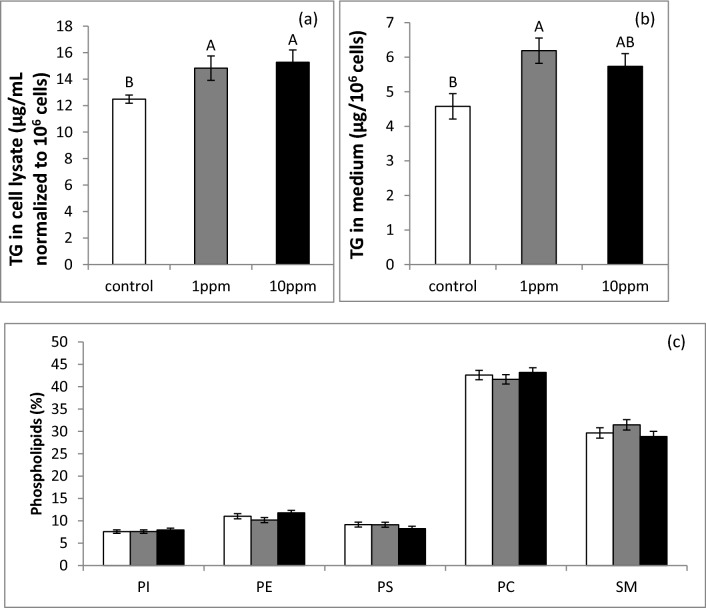
Effect of PLEE on TG content in the MEC (**a**), TG secretion into the medium (**b**), and MEC membrane composition, characterized by phosphatidylinositol (PI), phosphatidylethanolamine (PE), phosphatidylserine (PS), phosphatidylcholine (PC) and sphingomyelin (SM) (**c**). MEC were incubated without (control, white) or with 1 (grey) or 10 (black) ppm PLEE for 24 h; n = 4 for each replicate in each treatment. Different letters indicate significant difference at *P* ≤ 0.05.

The effect of PLEE on fat production and secretion into the medium was determined. Cells were harvested and medium was collected after 24 h of treatment with 1 or 10 ppm PLEE. Triglyceride (TG) content and phospholipid composition were determined by HPLC. PLEE treatment significantly increased the TG content in the cell, by 18–22% (Fig. 4a; *P* = 0.044), and in the medium for 1 ppm by 35% (Fig. 4b; *P* = 0.012) compared to controls. In addition, membrane phospholipid composition was determined, to assess membrane stability and the probability of lipid droplet size modifications due to enhancement of fusion events between intracellular lipid droplets. Phosphatidylcholine and sphingomyelin were the major phospholipids in the MEC membranes, accounting for approximately 40 and 30%, respectively, of all membrane phospholipids. The treatment did not affect the composition of the membrane phospholipids (Fig. 4c).

#### PLEE treatment alters protein secretion but not intracellular content by MEC

**Figure 5:**
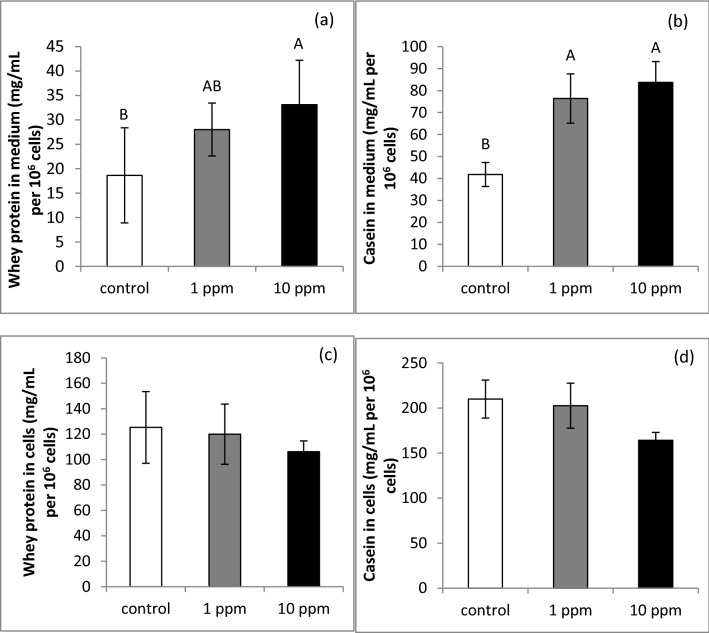
Effect of PLEE on intracellular content and secretion of whey protein and caseins. MEC were incubated without (control, white) or with 1 (grey) or 10 (black) ppm PLEE for 24 h. Whey protein and casein contents were determined in the medium to assess secretion ((**a**,**b**), respectively) and in the cell lysate, normalized to 1 million cells ((**c**,**d**), respectively); n = 4 for each replicate in each treatment. Different letters indicate significant difference at *P* ≤ 0.05.

We investigated the effect of PLEE on MEC protein synthesis and secretion in cell culture. After incubation without (control) or with 1 or 10 ppm PLEE, cells were harvested and medium was collected to determine the content and secretion of whey protein and caseins by HPLC. PLEE increased whey protein content in the medium in a dose-responsive manner, by 50 and 77% for 1 ppm and 10 ppm, respectively (Fig. 5a; *P* = 0.009), and casein protein content by 82 and 100%, respectively (Fig. 5b; *P* = 0.01), compared to controls. However, whey protein (Fig. 5c; *P* = 0.82) and casein (Fig. 5d; *P* = 0.24) contents in the cells did not differ between treatments.

#### PLEE treatment increases lactose production and secretion by MEC

**Figure 6:**
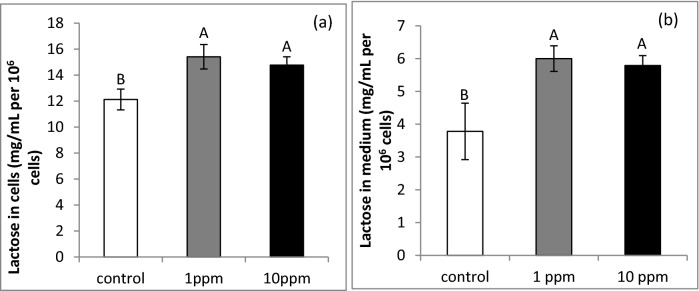
Lactose content, normalized to 1 million cells, in the cell lysate (**a**) and lactose secretion to the medium (**b**) of MEC incubated without (control, white) or with 1 (grey) or 10 (black) ppm PLEE for 24 h; n = 4 for each replicate in each treatment. Different letters indicate significant difference at *P* ≤ 0.05.

PLEE treatment significantly increased lactose content in the cells by 22 and 27% for 1 and 10 ppm PLEE, respectively (Fig. 6a; *P* = 0.02), and in the medium by 53 and 59%, respectively (Fig. 6b; *P* = 0.001) compared to controls.

### Effect of PLEE on gene expression in MEC

**Figure 7:**
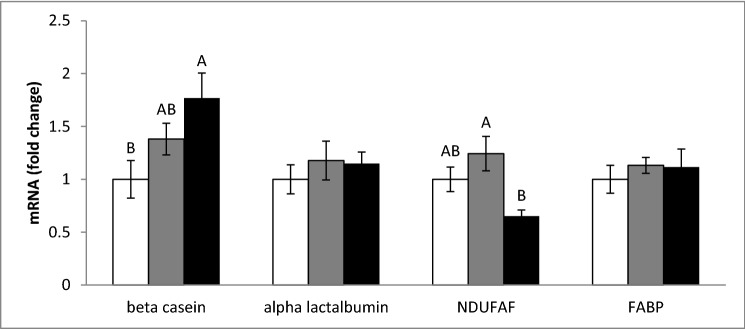
MEC were incubated without (control, white) or with 1 (grey) or 10 (black) ppm PLEE for 24 h and the mRNA expression of enzymes in the production chain of milk constituents and mitochondrial activity was assessed; NDUFAF3, NADH:ubiquinone oxidoreductase complex assembly factor 3; FABP, fatty acid-binding protein; n = 4 for each replicate in each treatment. Different letters indicate significant difference at *P* ≤ 0.05.

The effect of PLEE on the expression of genes encoding enzymes in the production chain of lactose, fat and protein, and a marker for mitochondrial activity—NADH:ubiquinone oxidoreductase complex assembly factor 3 (NDUFAF3), a key enzyme in the oxidative phosphorylation chain—was determined. Treatment with 10 ppm PLEE increased β-casein gene expression by 1.75-fold compared to controls (Fig. 7; *P* < 0.05). The expression of NDUFAF3 was marginally elevated by the 1 ppm treatment, and reduced by the 10 ppm treatment, resulting in a significant difference only between the 1 and 10 ppm treatments. The gene-expression levels of α-lactalbumin, a key enzyme in lactose biosynthesis, and of fatty acid-binding protein (FABP) were not modified by the PLEE treatment.

### PLEE increases mitochondrial count and activity in MEC

**Figure 8:**
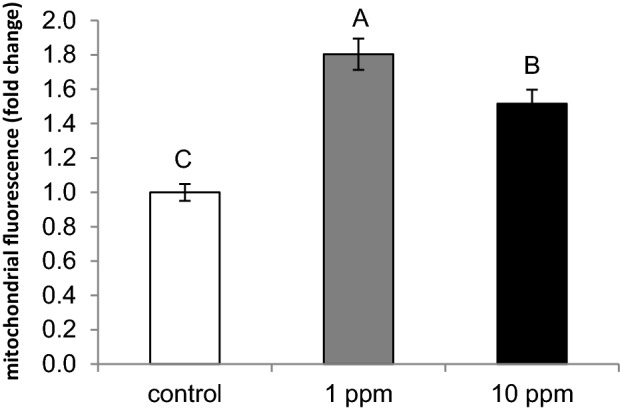
MEC were incubated without (control, white) or with 1 (grey) or 10 (black) ppm PLEE for 24 h and mitochondria were quantified by monitoring the fluorescence intensity after MitoTracker deep red staining. Results are presented as fold change compared to controls. All cells were imaged using identical exposure times for each staining; n > 110 cells in each treatment. Different letters indicate significant difference at *P* ≤ 0.05.

Cellular metabolic status can be determined by mitochondrial number, independent of the cell cycle. Therefore, MitoTracker red stain was used to evaluate the number of mitochondria in MEC incubated for 24 h without (control) or with 1 or 10 ppm PLEE. PLEE treatments increased mitochondrial amounts by 80 and 51%, respectively, compared to controls (Fig. 8; *P* < 0.0001).

**Figure 9:**
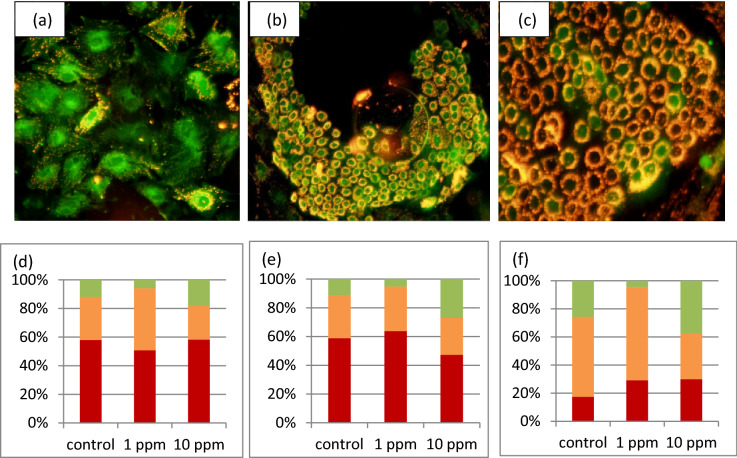
Three mitochondrial membrane phenotypes are presented: (**a**) green (low potential), (**b**) orange (moderate potential), and (**c**) red (high potential). MECs were incubated with 0 (control), 1 or 10 ppm PLEE for 24 h. The different phenotypes’ distribution at 0 h (**d**), 1 h (**e**) and 2 h (**f**) of H_2_O_2_ stress is presented in plots. All cells were imaged using identical exposure times for each staining. Each treatment reflects n > 600 cells.

To assess the role of polyphenols in protection against pro-oxidant challenge, MEC were treated with 0 (control), 1 or 10 ppm PLEE and then exposed to hydrogen peroxide (H_2_O_2_) for 2 h. Cells were stained with 5,5′,6,6′-tetra-chloro-1,1′,3,3′-tetraethylbenzimidazolyl carbocyanine iodide fluorescent probe (JC-1) before the exposure (Fig. 9d), and after 1 h (Fig. 9e) and 2 h (Fig. 9f) of H_2_O_2_ exposure. JC-1 distinguishes mitochondria with impaired membrane potential (green; Fig. 9a) from those with high (red; Fig. 9c) and intermediate (orange; Fig. 9b) membrane potential. Images were taken by fluorescence microscopy.

At time zero (Fig. 9d), PLEE treatment did not affect the distribution of high (*P* = 0.69), impaired (*P* = 0.48), or intermediate (*P* = 0.1) membrane potential. Control MEC exposed to 2 h of H_2_O_2_ without preincubation with PLEE showed an increasing proportion of cells with impaired mitochondrial membrane potential (from 11.8 to 25.6%; Fig. 9f). Preincubation with 1 ppm PLEE did not change the percentage of cells with damaged mitochondria compared to time 0 (*P* = 0.224). In MEC treated with 10 ppm PLEE, the proportion of cells with impaired membrane potential increased from 18% at time 0 to 37.5% 2 h after exposure to H_2_O_2_. Moreover, while exposure to H_2_O_2_ for 2 h adversely affected control cells with high membrane potential, from 57 to 17% (*P* < 0.001), no significant changes were observed after 1 h of exposure (Fig. 9e).

### PLEE treatment alters oxygen-consumption rates in MEC

**Figure 10:**
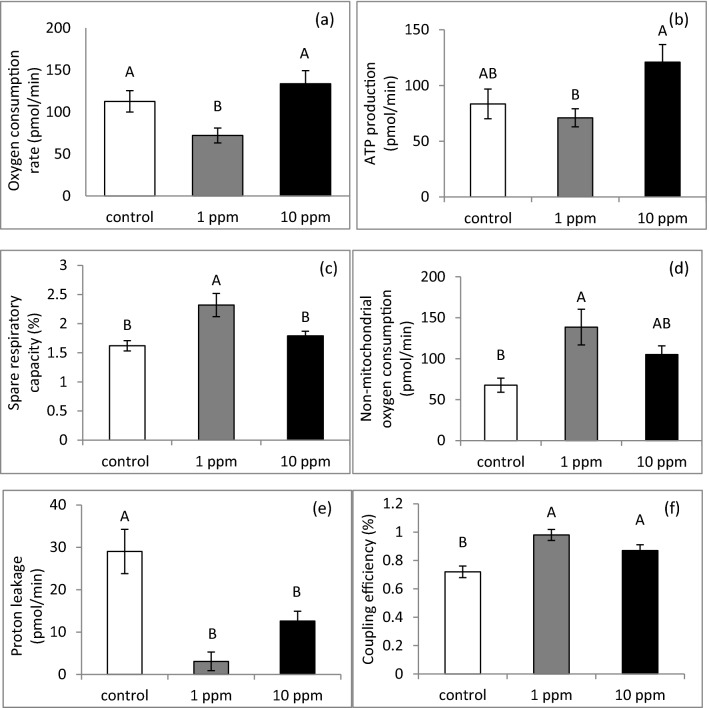
MEC were incubated without (control, white) or with 1 (grey) or 10 (black) ppm PLEE for 24 h and subjected to oxygen-consumption assay. Oxygen-consumption rate was measured for basal respiration (**a**). (**b**) ATP production, (**c**) spare respiratory capacity, (**d**) non-mitochondrial oxygen consumption, (**e**) proton leakage, (**f**) coupling efficiency; n = 5 for each replicate in each treatment. Different letters indicate significant difference at *P* ≤ 0.05.

MEC incubated without (control) or with 1 or 10 ppm PLEE were analysed for oxygen-consumption rate. Coupling efficiency, non-mitochondrial oxygen consumption and ATP production were calculated by Wave software (2.6.1.53 version), according to the manufacturer’s guidelines. Treatment with 1 ppm PLEE reduced oxygen-consumption rate compared to control and to the 10 ppm treatment (Fig. 10a; *P* < 0.01). Accordingly, mitochondrial ATP production was higher in cells treated with 10 vs. 1 ppm PLEE (Fig. 10b; *P* < 0.05). Spare respiratory capacity (Fig. 10c) and the maximal possible oxygen-consumption rate were measured after addition of carbonyl cyanide-4-(trifluoromethoxy) phenylhydrazone (FCCP), which artificially imposes supreme ATP demand but disperses the proton gradient and thus blocks conversion of ADP to ATP. While no differences were observed between PLEE treatments (*P* = 0.12) in maximal oxygen-consumption rate, the spare respiratory capacity in the 1 ppm treatment was 29.6 and 43.2% higher than in the 10 ppm treatment and controls, respectively (Fig. 10c; *P* < 0.01). At the end of the experiment, rotenone and antimycin A were added as inhibitors of respiratory complexes I and III, respectively, to determine the non-mitochondrial oxygen consumption. MEC treated with 1 ppm PLEE had significantly enhanced non-mitochondrial oxygen consumption compared to control MEC (Fig. 10d; *P* = 0.01). Despite the presence of oligomycin in the system, some mitochondrial respiration persisted due to the "uncoupling", which was expressed as proton leakage and coupling efficiency. Proton leakage was markedly decreased by PLEE, most notably by the 1 ppm treatment (Fig. 10e; *P* < 0.001). Consequently, the coupling efficiency in the respiratory chain and ATP synthase were significantly more advanced in PLEE-treated vs. control MEC (Fig. 10f; *P* < 0.001).

## Discussion

We investigated the direct effect of lentisk extract on MEC with a focus on production traits. PLEE positively affected production and metabolism of primary bovine MEC. In particular, enhanced lipid production was concomitant with increased diameter of intracellular lipid droplets (the milk fat globule precursors); casein secretion was elevated; lactose synthesis and secretion was enhanced, and mitochondrial activity was more resilient to pro-oxidant load, as expressed by antioxidant activity, coupling efficiency and spare respiration capacity.

In farm animals, the utilization of exogenous antioxidants was studied in several aspects: improvement of milk components and quality for human health^29,30^, vascular homeostasis during prooxidant challenge^31^, incidence of mastitis^32^ semen quality^33^, plasma redox status^34^ and weight gain of bull calves^35^. However, no cellular mechanism was investigated for these production traits till now.

We first wanted to explore how the phenolic compounds in PLEE interact with MEC in culture; several previous lines of evidence have suggested that the effect of phenolic compounds on cells can be mediated by their coating of, and incorporation into the plasma membrane, as found in erythrocytes^36^, endothelial cells^37^, rat cerebral membranes^38^ and semen cells^33^. In the present study, indeed, using confocal fluorescence microscopy, we clearly demonstrated the presence of PLEE polyphenols near the plasma membrane of MEC, in the cytoplasm, and around and in the nucleus (Fig. 1). PLEE’s presence in all main cellular domains indicates its potential involvement in intracellular biochemical activities.

Various antioxidants have been shown to have cytoprotective effects against oxidative stress. However, most of these studies were conducted on cell lines of mammary epithelium with limited capacity to synthesize and secrete milk constituents. For instance, exposure of a MAC-T line to resveratrol enhanced the expression of multiple antioxidant genes under normal and oxidative conditions via activation of Akt and ERK signalling pathways^16^. Moreover, the damage to MEC by activated neutrophils was totally inhibited by exposure of MAC-T cells to 50 µM catechin^39^. In primary culture of bovine MEC, treatment with tea polyphenols protected bovine MEC against oxidative stress by scavenging ROS and upregulating the expression of antioxidants and detoxifying enzymes^17^. In agreement with those results, we found a ninefold increase in the antioxidant capacity of MEC treated with PLEE (Fig. 2). Nevertheless, the mechanisms underlying the regulation of phenolic compounds and MEC production are not well understood.

In the present study, we used lipid droplet size as a proxy for fat production by MEC. PLEE treatments (both 1 and 10 ppm) increased lipid droplet size by 20% compared to controls. In general, lipid droplet size is positively correlated with cellular triacylglycerol (TG) content^40,41^, reduced lipolysis, and elevated capacity for lipogenesis^42^. Another mechanism which can contribute to lipid droplet size is fusion, which is tightly associated with cellular phospholipid composition^42^. To determine the dominant mechanism in the increase in lipid droplet size, we quantified the weight, content and composition of phospholipids, as the main membrane component altering membrane stability^43^ and potentially modulating lipid droplet fusion^44^. We found no significant change in MEC phospholipid composition among treatments, suggesting that the increase in lipid droplet size under the PLEE treatment is not driven by membrane instability and fusion but, most likely, by greater accumulation of TG in the cell. We extended our test by measuring the lipid-secretion capacity of the MEC and found an average enhancement of 35% in cells treated with 1 ppm PLEE compared to controls (Fig. 4).

While cellular whey protein and casein contents did not differ with PLEE treatment, amounts of secreted whey protein and casein were approximately twofold greater in the 10 ppm PLEE treatment compared to the control (Fig. 5). Although the mechanism is not clear, the increase in casein content was in accordance with an increase in β-casein gene expression. β-Casein is one of the major compounds secreted by MEC, accounting for 24–28 g protein per L of milk^45^, and found to be associated with milk and protein yields in Holstein cows^46^. The fact that greater protein secretion occurred under the PLEE treatment may be attributed to greater non-mitochondrial oxygen consumption recorded in the treated MEC as the protein-folding process prior to secretion occurs in the endoplasmic reticulum requires oxygen^47^. We assume that PLEE increased the cell’s oxygen consumption, which in turn increased endoplasmic reticulum activity, manifested by greater casein secretion.

PLEE increased cellular lactose content and elevated its secretion (Fig. 6), but it was not associated with any changes in α-lactalbumin gene expression. Lactose synthesis in MEC is a complex process, starting in the endoplasmic reticulum and continuing in the Golgi apparatus. The enzymes participating in this process are not always regulated in a coordinated manner. For instance, in bovine MEC cultured with 5–10 mmol/L glucose, the mRNA expression of B4GALT, the enzyme that transfers galactose to form lactose, and of lactose synthase was elevated, but that of α-lactalbumin was unchanged^48^. On the other hand, lactose synthesis can be allosterically modulated by the availability of its precursors. Two moles of glucose are required for every mole of lactose. The glucose available for this process is tightly associated with the redox status of the cell, because it can be used to generate NADPH in the pentose phosphate pathway. NADPH is a reducing agent, required for the reduction of oxidized glutathione to reduced glutathione. Therefore, increased cellular ROS production might enhance the utilization of glucose for NADPH production as a substrate for ROS quenching with oxidized glutathione by glutathione reductase^49^. In light of this, we suggest that PLEE has a glucose-sparing effect, hence releasing more glucose for lactose synthesis.

Taken together, we report the combined effects of PLEE on lipid, lactose, whey protein and casein production, indicating a general effect on productivity, even though we are aware that the improved production traits were not always in agreement with gene-expression levels, suggesting a general bioenergetic effect, presumably through mitochondrial quantity and functionality. Accordingly, exposing MEC to 10 ppm PLEE increased basal respiration, which was expressed in elevated ATP production (Fig. 10). On the other hand, treatment with 1 ppm PLEE did not change basal respiration. However, both treatments reduced proton leakage and increased coupling efficiency. These results imply that the treated cells utilize oxygen more efficiently, and synthesize more ATP without changing their oxygen-consumption rate. Consequently, it is suggested that the greater production of milk constituents by the PLEE-treated cells is due to a greater ATP production capacity and most probably, less inhibition by ROS in the metabolic and production pathways.

The main ROS producers in the cell are the mitochondria, with enhanced ATP production being coupled with higher ROS production^50^, and the endoplasmic reticulum during protein folding^41^. To further investigate the mitochondrial resistance to pro-oxidant load, we exposed MEC to H_2_O_2_ as in Flora et al.^51^. We found that the mitochondria of the PLEE-treated cells could maintain high membrane potential, even under this exogenous oxidative challenge. The inner membrane of mitochondria in the PLEE-treated cells maintained higher potential and durability compared to controls, suggesting that lentisk polyphenols directly protect the energy-production mechanism of MEC from pro-oxidant load. These findings are in line with the previously reported antioxidant capacity of various polyphenols^52^. In particular, gallic acid, a main component in the lentisk extract^53^, is a strong electron donor to ROS^54^. For instance, in mouse, gallic acid provided indirect protection by elevating the hepatic levels of glutathione peroxidase and catalase, thereby potentially providing direct and indirect protection against the pernicious effects of H_2_O_2_^55^. Changes in the expression of genes encoding antioxidant enzymes such as HO-1, Xct, Txnrd1, and NQO-1 through Nrf2 occur in a timeframe of 4–25 h^16^, and this was therefore not assessed in the present study, where exposure to oxidative challenge only lasted 2 h.

It should be noted that the PLEE polyphenols entered the MEC in a dose-dependent manner, as autofluorescence was more intense in the 10 ppm compared to 1 ppm treatment, however this was not necessarily correlated with cell function. Moreover, 2 h after exposure to H_2_O_2_, the proportion of cells with impaired membrane potential increased in MEC treated with 10 ppm PLEE, but not with 1 ppm-treated cells. Furthermore, 10 ppm-treated cells had higher ATP production and less spare respiratory capacity than those treated with 1 ppm. Taken together, these results imply that 10 ppm PLEE can be somewhat toxic to the cells for specific energetic processes, while remaining beneficial to other cellular processes.

The effect of exogenous phenolic compounds on milk production has mainly been studied in vivo, with its polyphenols administered as part of the diet. In vivo, dairy goats browsing on lentisk produced milk with more fat and protein and lower urea content compared to their counterparts fed a hay-based diet with undetectable tannin content^26^. Similar results were obtained when *Lotus corniculatus* was used as a phenolic compound source to feed dairy ewes^56^ and cows^57^; *L. corniculatus* elevated milk protein content and reduced milk urea content^28,58^. These findings are explained by the protein-binding activity of tannins, protecting the protein from bacterial degradation and hence increasing protein flow in the duodenum, and its availability for the synthesis of milk proteins^52^. However, low contents of dietary phenolic compounds such as quebracho tannins^59^ and *Acacia mearnsii* tannin extract^60^ supplemented to dairy cows affected milk fatty acid composition, which could probably be attributed to a systemic effect of the phenolic compounds after their absorption into the bloodstream, and not to a local effect in the gastrointestinal tract.

In summary, the assumption that dietary phenolic compounds exert a systemic effect post-absorption is supported by the finding that dietary phenolic compounds are absorbed into the bloodstream and transported into milk^61^, indicating that the mammary cells are directly exposed to these chemical compounds. The results of the current study shed light on the direct effect of phenolic compounds on MEC, and how exposure to these compounds modulates energy status of the cells, and probably utilization of reducing agents and nutrients (such as NADPH and glucose) for production instead of for damage control. Supplementation of lentisk polyphenols to the culture medium enhanced energy production, which was directed to lipid, protein and lactose production instead of oxidative-damage regulation. Such knowledge could provide the foundation for developing new profitable and sustainable nutritional strategies to enhance mammary gland productivity while coping with stress.

## Materials and methods

### Chemicals and reagents

DMEM/F12, fetal bovine serum (FBS), penicillin, streptomycin, amphotericin B, l-glutamine solution and trypsin–EDTA solution C were purchased from Biological Industries (Beit Haemek, Israel). Bovine insulin, hydrocortisone, ovine prolactin, bovine serum albumin (BSA) solution, hyaluronidase, DNase I and heparin were purchased from Sigma Aldrich Israel (Rehovot, Israel). Collagenase type II was purchased from Worthington Biochemical Corporation (Lakewood, NJ, USA).

For lipid extraction, methanol and chloroform (both analytical reagent grade) were purchased from Bio-Lab (Jerusalem, Israel). For HPLC analysis, chloroform and ethanol (used at 97:3 v/v, both analytical reagent grade) and methanol (HPLC grade) were purchased from Bio-Lab. TG (triglyceride triolein, purity > 99%) was purchased from Supelco (Bellefonte, PA, USA). Phospholipid standards were from Sigma Aldrich Israel and consisted of the following: PE (1,2-dioleoyl-sn-glycero-3 phosphoethanolamine, 10 mg phospholipid per mL CHCl_3_, purity 99%), PI (l-α phosphatidylinositol ammonium salt, from bovine liver, purity 98%), PS (1,2-dioleoyl-sn-glycerol-3-phospho-l-serine sodium salt, purity 95%), PC (1,2-dioleoyl-sn-glycero-3-phosphocholine, purity ≥ 99%) and SM (sphingomyelin; from bovine brain, purity 97%). Commercial standard mixes of phospholipids and TG were injected to determine retention times.

### Experimental design

Primary MEC were plated at 50,000 cells per well in 6-well plates on glass cover slips for MitoTracker red, Nile red or JC-1 staining and MEC autofluorescence determination; at 150,000 cells per 60-mm plastic dish for cellular lipid, protein and lactose extraction, RNA extraction, and antioxidant capacity determination; and at 28,000 cells in XF24 cell-culture microplates (Seahorse Bioscience, North Billerica, MA, USA) for cellular metabolic flux analyses. Cells were grown in plastic culture dishes with DMEM/F12 supplemented with 10% FBS, 100 U/mL penicillin, 100 μg/mL streptomycin, 0.25 μg/mL amphotericin B, 1 μg/mL insulin and 0.5 μg/mL hydrocortisone. When plates reached to 80% confluence, which was approximately 3 days post-plating, medium was replaced with DMEM/F12 without serum, containing 0.15% (w/v) free fatty acid-free BSA and insulin (1 μg/mL), hydrocortisone (0.5 μg/mL) and prolactin (1 μg/mL) for 48 h to induce milk lipid, protein and lactose synthesis. Then cells were treated for 24 h with treatment medium that contained 1 ppm or 10 ppm of a 70% ethanolic extract of *P. lentiscus* foliage, all dissolved in DMEM/F12 supplemented with insulin (1 μg/mL), hydrocortisone (0.5 μg/mL), prolactin (1 μg/mL) and 0.1 M oleic acid (C18:1). Doses of PLEE used in the present study were selected according to the results of preliminary dose response experiment with 0, 1, 10 and 100 ppm of PLEE. Doses were chosen according to their toxic effect on live cell percentage after 24 h of treatment and according to solubility in the medium. Compared with control (0 ppm) no change in cells live cells number was visualized under 1 and 10 ppm.

After 24 h, cells were harvested for cell counting, lipid extraction, protein and lactose quantification and antioxidant capacity determination, or for staining of intracellular lipid droplets or mitochondria, or JC-1 staining. The cells were not harvested for counting for real-time PCR; cells were harvested at the end of the oxygen-consumption assay for cell counting and normalization.

#### Primary culture

Primary culture of MEC was isolated from mammary biopsies according to a protocol established in our laboratory^44,62^ with slight changes. Briefly, udder tissue was collected from three lactating cows at Rahat commercial slaughterhouse. Tissues were collected after cows were commercially slaughtered by a certified slaughterhouse worker, and after veterinarian inspection, and transferred to the laboratory. Only udders with normal structure from lactating cows were used. Tissues were immediately submerged in DMEM/F12 medium supplemented with 10% (w/v) FBS, 100 U/mL penicillin, 100 μg/mL streptomycin, 0.25 μg/mL amphotericin B, 1 μg/mL insulin and 0.5 μg/mL hydrocortisone (growth medium). Tissue (10 g) was digested by shaking in 100 mL of growth medium supplemented with collagenase (1 mg/mL), hyaluronidase (1 mg/mL) and heparin (0.02 mg/mL) at 100 rpm for 3 h combined with two controlled breaks to enrich the suspended fraction, at 37 °C. After incubation, the suspension was filtered through a metal mesh (250 μm), and the filtrate was centrifuged at 350×*g* for 5 min. The sediment was treated with trypsin–EDTA and 0.04% (w/v) DNase. Cells were washed with growth medium supplemented with heparin and treated with DNase, filtered using a 100-μm cell strainer (BD Falcon, Bedford, MA, USA) and then washed with the growth medium. Cells were grown in plastic culture dishes with DMEM/F12 supplemented with 10% FBS, 100 U/mL penicillin, 100 μg/mL streptomycin, 0.25 μg/mL amphotericin B, 1 μg/mL insulin and 0.5 μg/mL hydrocortisone.

#### Plant material collection, extraction and fractionation

Leaves from *P. lentiscus* were randomly collected from Ramat Hanadiv Nature Park (south of Carmel Heights, 32° 33′ N, 34° 56′ E) during the summer and spring. The lentisk leaf extract was prepared according to Azaizeh et al.^53^. Briefly, the leaves were dried at 50 °C for 24 h, ground and stored for further analysis at room temperature. Powdered leaf tissue samples (10 g each) were incubated with 100 mL of 70% ethanol at 35 °C for 24 h. The crude ethanol extract was filtered and evaporated under vacuum (Rotorvapor Hie-VAP; Hiedolph, Germany) at 45 °C to remove the ethanol and water. The extraction yield was calculated as gram extract per gram dry matter (plant leaves), and the dried extracts were stored at − 20 °C. On treatment day, dried extract was diluted with double-distilled water to 1 and 10 ppm and named PLEE (polyphenols from lentisk ethanolic extract). Dried plant extract of lentisk was analysed for phenolic compounds using HPLC as previously described^53^. Briefly, for hydrolysis, 250 mg of dried extract was dissolved in 25 mL of 1% HCl, and incubated at 70 °C for 8 h with shaking at 100 rpm. The presence of polyphenols and flavonoids in the hydrolysed and non-hydrolysed solutions was determined using reversed-phase HPLC analysis with binary gradient elution on a Thermo Scientific Finnigan Surveyor system equipped with a PDA Plus Detector (220–450 nm) (Thermo Fisher Scientific, Waltham, MA, USA). As recently published by Hadaya et al.^29^, the chromatographic separation was performed on a Gemini 5µ C6-Phenyl 110 Å column (250 × 4.60 mm) (Phenomenex, Torrance, CA, USA) at 30 °C. The mobile phase consisted of 0.1% acetic acid in water (A) and 0.1% acetic acid in methanol (B), and the run was programmed for 35 min. The elution conditions were: 0 to 5 min, 25% B; 5 to 15 min, 25 to 50% B; 15 to 20 min, 50% B; 20 to 29 min, 50 to 25% B; 29 to 35 min, 25% B. The flow rate was 1.0 mL/min and the injection volume was 15 µL. Standards for tyrosol, luteolin-7-O-glycoside, apigenin-7-O-glucoside, quercetin-7-O-rutinoside, luteolin, quercetin, gallic acid and rutin were from Sigma Aldrich Israel; and for hydroxytyrosol, oleuropein, chlorogenic acid, catechin and myricetin, from Cayman Chemicals (Ann Arbor, MI, USA). The polyphenols of the ethanolic phospholipid extract consisted mainly of galloyl derivatives (63.6%), flavonol glucosides (28.6%) and catechin (7.8%).

### Lipid extraction and analysis

After the 24-h treatment with PLEE, total lipids were extracted from harvested cells with trypsin (0.05%), washed with phosphate buffered saline (PBS) and stored at − 20 °C until lipid extraction as previously described^62^. Briefly, a 5 mL of chloroform:methanol solution (2:1, v/v) was added to each sample. After incubation at room temperature, 1 mL of double-distilled H_2_O was added. After overnight incubation with cold extraction at 4 °C, the upper phase was removed, and the lower phase was filtered through glass wool. Samples were then evaporated under a nitrogen stream at 65 °C, diluted in 100 μL chloroform:methanol (97:3, v/v) and stored at − 20 °C until injection for HPLC analysis. Separation of polar and neutral lipids was performed on a silica column (Zorbax RX-SIL, 4.6 × 250 mm, Agilent Technologies, Santa Clara, CA, USA) by HPLC (HP 1200, Agilent Technologies) with an evaporative light-scattering detector (1200 series ELSD, Agilent Technologies). The column was heated to 40 °C, flow was 1 mL/min, and injection volume was 20 μL. The ELSD was heated to 65 °C, nitrogen pressure was 3.8 bars, the filter was set to 5, and gain (sensitivity) was set to 4 for the first 14 min, then changed to 12 until 21 min, and then to 7 until the end of the run to enable detection of differently abundant lipid components. The separation protocol consisted of a gradient of dichloromethane, methanol:ammonium mix (99:1, v/v) and double-distilled water. The separation process was managed by ChemStation software (Agilent Technologies), which permitted acquisition of data from the ELSD detector. The separated lipids were identified using external standards (Sigma Aldrich, Israel). Quantification was performed against external standard curves and expressed as µg/per 10^6^ live cells or as weight % out of the sum of phospholipids (µg) in the sample. Live cell number was determined with a haemocytometer after 5 min of Trypan blue staining.

### Protein extraction and analysis

After the 24-h treatment with PLEE, cells were harvested with trypsin (0.05%), washed with phosphate buffered saline (PBS) and stored at − 20 °C until protein extraction, and 0.5 mL of medium were collected for further analysis. Cells and medium were lysed by 4 rotations of freezer and thaw cycles followed by 30 min of sonication. Then, cells were centrifuged in 350 g for 5 min and 0.5 mL of upper phase was collected and filtered in 0.45 µm (PES) and stored in − 20 °C until injection for HPLC analysis. Separation of caseins and whey was performed on a silica column (Zorbax Eclipse XDB-C18, 4.6 × 150 mm, Agilent Technologies, Santa Clara, CA, USA) by HPLC analysis with binary gradient elution on a Thermo scientific Finnigan Surveyor system equipped with a PDA plus Detector (220–450 nm) (Thermo Fisher scientific Inc., Waltham, MA, USA). Protein content was determined at 220 nm. The elution gradient was 1 mL/min at 30 °C for 30 min; the injected volume was 20 µL. The mobile phase consisted of mixed acetonitrile:water:trifluoroacetic acid (100:900:1, v/v/v, for solvent A and 900:100:1, v/v/v for solvent B). A run consisted of 1–4 min of 100% solvent A; 4–20 min, 100 to 25% solvent A; 20–24 min, 25% solvent A; 24–26 min, 25 to 100% solvent A; 26–30 min, 100% solvent A. Identification and quantification were determined by constructing a calibration curve of an external standards of known protein concentration dissolved in 50 mM phosphate buffer (pH 6.7). Standard curves for α-casein (≥ 70%), β-casein (≥ 98%), α-lactalbumin and β-lactoglobulin (Sigma Aldrich, Israel) created after injection in concentrations of 50, 100, 200, 500, 1000, 2000 and 5600 ppm. Identification of α-casein, β-casein and α-lactalbumin qualified as caseins (α- and β-casein) and whey protein (α-lactalbumin), with no identification to β-lactoglobulin in the samples. Calibration curve strength for caseins and whey protein were R^2^ = 0.94 and R^2^ = 0.96, respectively (example for chromatograms of standard, medium and cells are provided in Supplemented Data File 1).

### Lactose extraction and analysis

After the 24-h treatment with PLEE, cells were harvested with trypsin (0.05%), washed with phosphate buffered saline (PBS) and stored at − 20 °C until lactose extraction, and 0.5 mL of medium were collected for further analysis. Cells and medium were lysed by 4 rotations of freezer and thaw cycles followed by 30 min of sonication. Then, cells were centrifuged in 350 g for 5 min and 0.5 mL of upper phase was collected and filtered in 0.45 µm (PES) and stored in − 20 °C until injection for HPLC analysis. Lactose was separate on a silica column in a Rezex-ROA-acids H^+^ (8%) 150X78 mm column, as previously described by Tayeh et al.^63^. HPLC analysis with binary gradient elution on a Thermo scientific Finnigan Surveyor system equipped with a refractive index detector at 68 °C. Sulfuric acid (0.005 N) was used for elution at flow rate of 0.6 mL/min for 14 min; the injected volume was 20 µL. Standard curves for lactose (Sigma Aldrich, Israel) created after injection in concentrations of 50, 100, 200, 500, 1000, 2000 and 5600 ppm. Identification and quantification were determined by establishing a calibration curve of external standard of known lactose concentration dissolved in water. The calibration curve for lactose had an R^2^ = 0.99.

### Lipid droplet staining

Cells grown on glass cover slips were rinsed three times with phosphate buffered saline (PBS) and fixed with 4% paraformaldehyde in PBS for 20 min at 25 °C. Cover slips were rinsed four times with PBS and stained with Nile red (200 nM, Sigma, St. Louis, MO, USA) for 15 min. Then cover slips were rinsed three times with PBS and stained with 4′,6-diamidino-2-phenylindole (DAPI; Sigma) for 5 min. Cover slips were once again rinsed four times with PBS and mounted with fluorescence mounting medium (Dako, Carpinteria, CA, USA). Slides were visualized with an Olympus BX40 fluorescence microscope equipped with an Olympus DP73 digital camera using cellSens Entry software version 1.7 (Olympus). Lipid droplet diameter was measured using ImageJ software version 1.48 (NIH, Bethesda, MD, USA).

### Quantification of mitochondrial fluorescence

After treatment, cells were incubated in DMEM/F12 with 500 nM MitoTracker red FM immunostain (Cell Signaling Technology, Danvers, MA, USA) for 30 min at 37 °C. The cells were then fixed in ice-cold 100% methanol for 15 min at − 20 °C and rinsed three times with PBS. Cells were mounted with fluorescence mounting medium (Dako), and slides were visualized with an Olympus BX40 fluorescence microscope equipped with an Olympus DP73 digital camera using cellSens Entry software version 1.7. Mitochondrial fluorescence in each cell was quantified by ImageJ software version 1.48 using the following formula: Corrected total cell fluorescence = Integrated density of selected cell − (Area of selected cell x Mean fluorescence of background readings).

### Mitochondrial membrane potential

After PLEE treatment on glass cover slips, cells were incubated with H_2_O_2_ to establish an in-vitro oxidative-stress model. Immediately before use, 30% H_2_O_2_ was diluted to 0.1 M stock using sterilized PBS. The 1 M stock was further diluted with cell culture medium to a final concentration of 0.5 mM H_2_O_2_ for 1 or 2 h. Then, to measure MEC mitochondrial membrane potential, cells were incubated for 10 min at 37 °C with 153 µM JC-1 (Enzo Life Sciences International, Plymouth Meeting, PA, USA). Cells were then rinsed three times with PBS and visualized with an Olympus BX40 fluorescence microscope equipped with an Olympus DP73 digital camera using cellSens Entry software version 1.7. Mitochondrial membrane potential fluorescence in each cell was qualified by ImageJ software version 1.48, based on the emitted fluorescence, and classified into three colors: red—high potential, orange—moderate potential and green—low potential. The different cell phenotypes within each treatment were further compared at different time points by one-way ANOVA.

### Confocal laser microscopy

After PLEE treatment on glass cover slips, cells were rinsed three times with PBS, and visualized and analysed in a confocal laser microscope (Leica TCS SP8) with X63 objective, and double excitation at 405 and 514 nm was used to visualize the autofluorescence of the polyphenols in washed primary culture of MEC.

### Determination of MEC antioxidant capacity

A LDCL assay was used to determine whether the PLEE treatments have the ability to bind and penetrate MEC and increase their oxidant-scavenging ability. The method is based on the generation of light-conjugated free-radical production. The ability of the treated MEC to quench light in the LDCL assay indicates whether the extracts have bound to the cells. Thus, lower LDCL values reflect samples with higher antioxidant capacity. The reaction cocktail contained Hank’s balanced salt solution, H_2_O_2_ and 1 mM cobalt chlorine, sodium selenite and luminol, in a total volume of 1 mL, to generate a constant flux of light due to H_2_O_2_ and cobalt-catalysed hydroxyl radical. A 40-µL aliquot of cells was added to the reaction mixture and analysed immediately in a Lumac type 2500 M luminometer for LDCL generation. The samples were measured for 12 min, and values were calculated according to a calibration curve as optical density equivalents to gallic acid concentration (R^2^ = 0.984).

### Gene expression

#### RNA extraction and cDNA synthesis

Total RNA was isolated from the primary culture of MEC by Gene Elute Mammalian Total RNA Miniprep Kit (Sigma Aldrich, Israel) according to the manufacturer’s instructions. The concentration and 260/280 nm optical density ratio of the RNA was determined by spectrophotometer (NanoDrop Technologies, Wilmington, DE, USA). RNA samples were kept at − 80 °C until further analysis. Total RNA (1 μg) was reverse-transcribed to produce cDNA using the qScript cDNA Synthesis Kit (Quanta Biosciences) according to the manufacturer’s instructions.

#### Real-time PCR analysis

Self-designed primers were produced using Primer-BLAST software (NCBI, http://www.ncbi.nlm.nih.gov/tools/primer-blast/index), based on cDNA sequences published in the NCBI database and validated by PCR-product sequencing. Primers were synthesized by Sigma Aldrich Israel according to the sequences, as indicated (Table 1).

**Table 1:** Primer sequences used for real-time PCR analysis.

Gene	Accession number	Sequence	Size (bp)	Reference
β-casein	NM_009972	F: AGAGGATGTGCTCCAGGCTA	230	Self design
		R: TAAGGAGGGGCATCTGTTTG		
α-Lactalbumin	NM_174378.2	F: TGTCTCTCGCTCCTGGTAGG	106	Self design
		R: ACCTCCGTAGCCCTTCAAGT		
FABP	NM_001078162.2	F: CCTTATCCGCCGCTTTATC	91	Self design
		R: TCTCCGTCAGCTTCCAGGTA		
DGAT-1	NM_174693.2	F: CGACTCCTGGAGATGCTGTT	116	Self design
		R: ATGCGGGAGTAGTCCATGTC		
NDUFAF3	NM_001046105.2	F: ACGAGCTGTATCAACGGACG	162	Self design
		R: AACCTACGTTCCACTGCACC		
β2-Microglobulin	NM_173893	F: CATCCAGCGTCCTCCAAAGAT	131	^64^
		R: CCCCATTCTTCAGCAAATCG		
18S	AF_176811	F: AGAAACGGCTACCACATCCA	169	^65^
		R: CACCAGACTTGCCCTCCA		

cDNA was mixed with the primers and platinum SYBR Green qPCR Supermix-UDG without ROX (Invitrogen Corporation, Carlsbad, CA, USA). A Mx3000P Real-Time PCR System (Stratagene, La Jolla, CA, USA) was used. Analysis was performed by MxPro software version 4.10 (Stratagene). Dissociation-curve analysis was performed after each real-time experiment to confirm the presence of only one product. The efficiency of the reaction and the initial mRNA quantity in the sample were determined using LightCycler 96 software version 1.1.0.1320 (Roche, Basel, Switzerland), and the ΔΔCt method was used to calculate the relative expression of each gene. The efficiency of the reaction and the initial mRNA quantity in the sample were determined using DART-PCR software version 1.0. Data were normalized to the geometrical mean of two housekeeping genes: *18S* and *β2-microglobulin*, and presented as fold change relative to the control treatment.

### Oxygen-consumption rate

Oxygen-consumption rate of live cells in a 24-well plate was measured in real time using a Seahorse Bioscience XF24 extracellular flux analyser. Cell number optimization was determined on 28,000 cells/mL which were seeded and grown for 24 h to 70–100% confluence before the metabolic flux analysis. Then, cell growth medium was replaced with XF assay medium (pH 7.4, Seahorse Biosciences) supplemented with 2 mM glutamine and 1 mM sodium pyruvate. Prior to the cell-respiration measurement, cells were incubated for 1 h at 37 °C without CO_2_. Basal oxygen consumption, maximal respiratory capacity and non-mitochondrial oxygen consumption were determined using the XF Cell Mito Stress Test Kit (Agilent Technologies, UK). The inhibitors of mitochondrial respiration, including oligomycin, carbonylcyanide p-trifluoromethoxyphenylhydrazone (FCCP) and rotenone/antimycin were auto-injected into the experimental wells after basal measurements. Oligomycin used to inhibit ATP synthase, FCCP used as a protonophore, Rotenone and antimycin blocked mitochondrial respiration of electron transport chain. Cell number determined by haemocytometer was used to normalize the oxygen-consumption rate values between wells (n = 4 for each treatment) and treatments.

### Scanning electron microscopy (SEM)

Primary MEC were fixed using the methanol method described by Talbot and White^66^. The sample was then dried in a critical point dryer (CPD-030, Bal-Tec/Leica, Wetzlar, Germany) and gold-coated in a sputter-coating unit (Quorum Technologies/Polaron, Laughton, UK). The sample was observed by low-vacuum SEM (JSM 5410 LV, Jeol, Tokyo, Japan). The SEM images were used to measure secretion area in the cell membrane.

### Statistical analysis

All statistical procedures were performed using JMP software version 12.0.1 (SAS Institute, Cary, NC, USA). Reported data are means ± SE. Dependent variables were checked for homogeneous variance by unequal variances in JMP software, and if the variance was not homogeneous, a Welch–ANOVA test was performed. Effects and comparisons between treatments were tested by ANOVA followed by LS Mean Tukey–Kramer HSD multiple-comparison test. The distribution of cell phenotypes based on mitochondrial potential membrane categories was compared by Chi square test. Significance probe was set to 0.05.

## Supplementary information

Supplementary Information.
