# Supplementary material for: Pistacia lentiscus extract enhances mammary epithelial cells’ productivity by modulating their oxidative status
Source: Sci Rep. 2020 Dec 2;10:20985. doi: 10.1038/s41598-020-78065-z (PMC7710751; doi:10.1038/s41598-020-78065-z)
Supplement: Supplementary file 1 — Supplementary Information. [file 41598_2020_78065_MOESM1_ESM.docx]

***Pistacia lentiscus* extract enhances mammary epithelial cells' productivity by modulating their oxidative status**

**O. Hadaya^a,b,*^, R. Bransi-Nicola^c^, Y. Shalev^a^, H. Azaizeh^c^, Z. Roth^a^, H. Muklada^b^, T. Deutch^b^, S.Y. Landau^b^, and N. Argov-Argaman^a^**

**Supplemented data file**

Figure 1 shows an example of chromatograms of 100 ppm beta casein standard (a), medium (b) and cells (c).


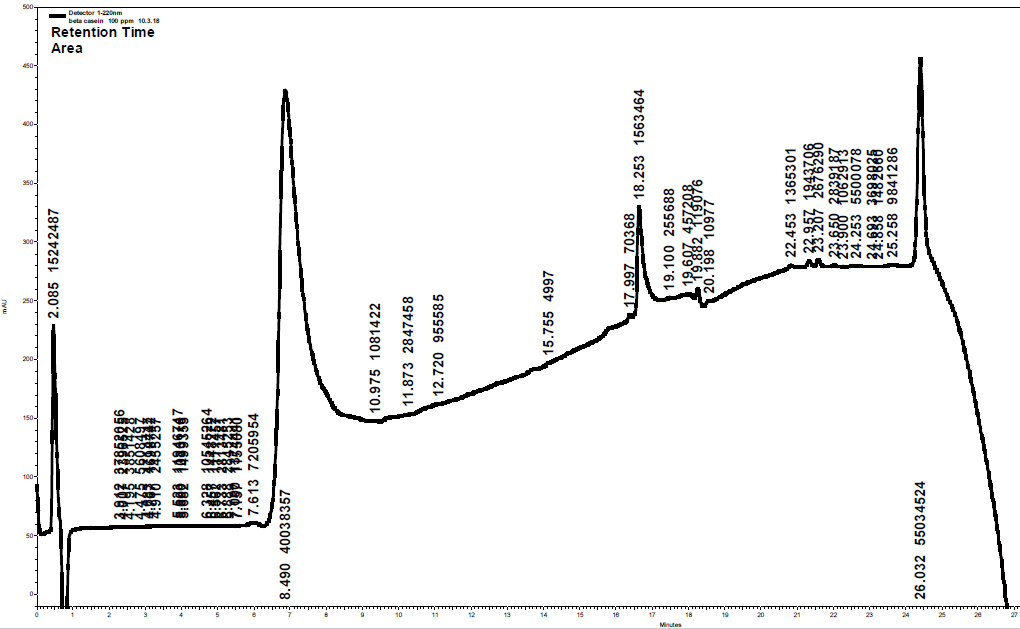


Figure 1a. Standard of beta casein in 100 ppm concentration. Retention time at 18.25 min.


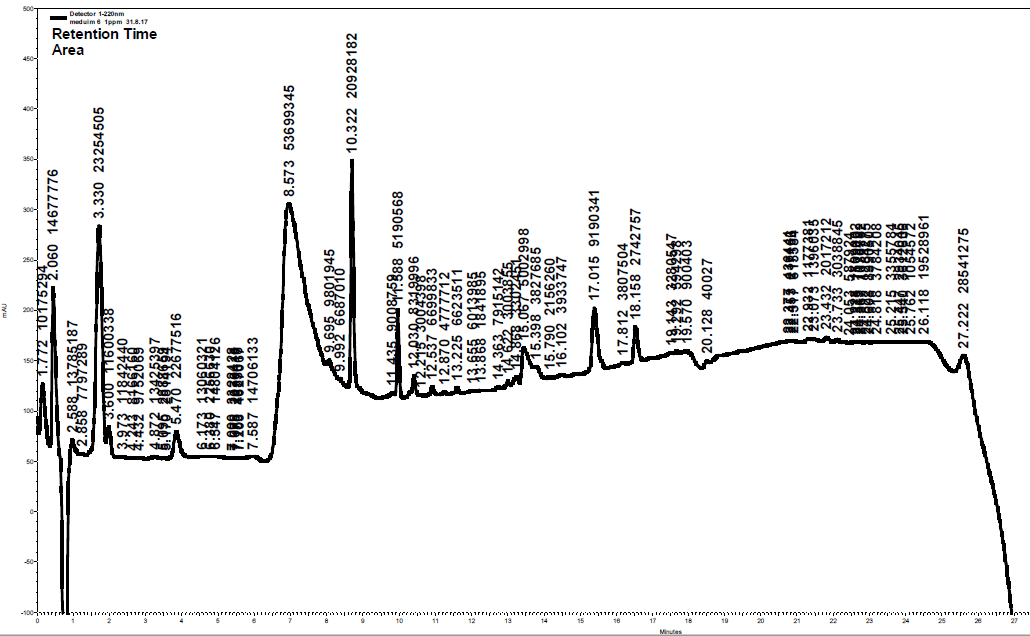
Figure 1b. An example of 1 ppm treatment of collected medium (beta casein peak on 18.15 min).


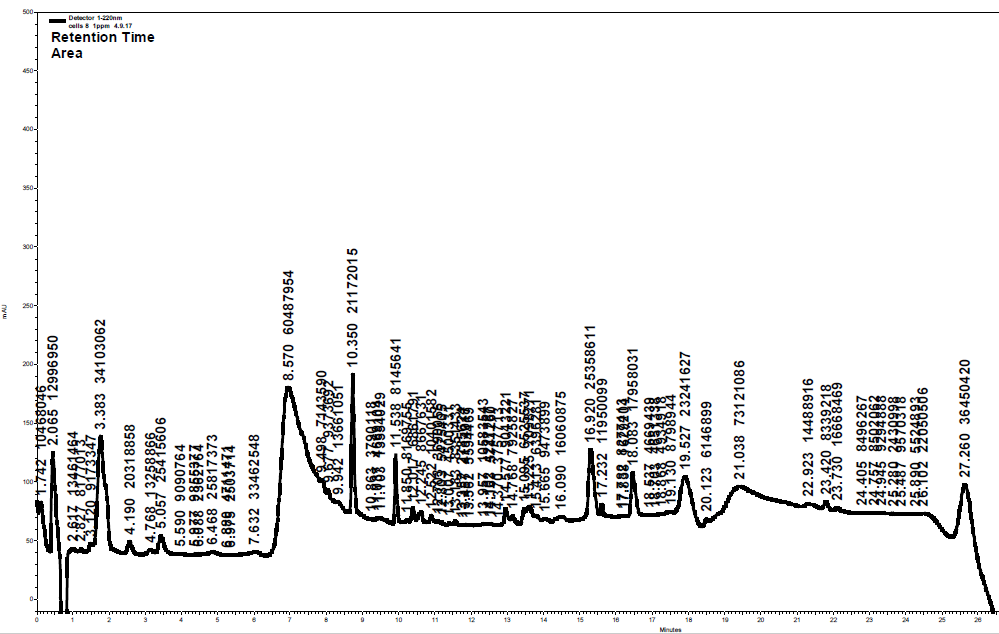


Figure 1c. An example of 1 ppm treatment of lysate cells (beta casein peak on 18.08 min).
